# Nonapeptide cell size differs between male morphs of the West African cichlid, *Pelvicachromis pulcher*

**DOI:** 10.1111/jfb.70306

**Published:** 2025-11-27

**Authors:** Adam R. Reddon, Douglas R. Wylie, Peter L. Hurd

**Affiliations:** ^1^ School of Biological and Environmental Sciences Liverpool John Moores University Liverpool UK; ^2^ Department of Biology University of Alberta Edmonton Alberta Canada; ^3^ Neuroscience and Mental Health Institute University of Alberta Edmonton Alberta Canada; ^4^ Department of Psychology University of Alberta Edmonton Alberta Canada

**Keywords:** alternative reproductive tactics, AVT, isotocin, oxytocin, rainbow krib, vasotocin

## Abstract

Alternative male morphs are found in many species of fishes. These morphs often differ in suites of social behaviours, such as aggression and territoriality, associated with alternative reproductive tactics. Such consistent morph‐typical behavioural profiles suggest common differences in underlying neuroendocrine mechanisms. The nonapeptide hormones oxytocin and vasotocin are linked to a wide range of behaviours, including aggression, mating behaviour and social attraction. These behaviours often differ between male morphs, suggesting that nonapeptides may mediate behavioural variation between morphs. We compared two morphs of a West African cichlid, *Pelvicachromis pulcher*, to test for differences in nonapeptide neuronal phenotypes. *P*. *pulcher* exhibit at least four distinct male colour morphs, of which the red and yellow morphs are the most common and have the best‐described social and reproductive behaviours. Red males tend towards breeding as harem holders, whereas yellow males breed monogamously or act as satellite males on the territories of red males. Here, we examine nonapeptide‐producing neurons in the preoptic area of the hypothalamus in the red and yellow morphs. We found that the more aggressive polygynous red morph has larger parvocellular oxytocin and vasotocin‐producing neurons, as well as larger gigantocellular oxytocin neurons, compared to the yellow morph. We did not find any association between the number of oxytocin or vasotocin cells and male morph. Our results suggest that the production of oxytocin and vasotocin in the preoptic area may play a role in determining morph‐typical behaviour among *P. pulcher* males.

## 1 INTRODUCTION

Individuals of a species will always show some variation in phenotype, which usually appears as a normal deviation from a species‐typical average (Bull, 1987). However, some species exhibit discrete morphs where different individuals of the same sex show a categorical distinction in phenotype (Mank, 2023; Oliveira et al., 2008; West‐Eberhard, 1986). Alternative morphs are more common in males and are often strongly associated with alternative reproductive tactics, such as territorial parental males and non‐territorial sneaker males (Taborsky & Brockmann, 2010: Neff & Svensson, 2013; Da Silva et al., 2025; but see Wang et al., 2024). For example, in lizard species such as *Uta stansburiana* and *Urosaurus ornatus*, males feature polymorphic throat badges associated with differing reproductive strategies (Moore et al., 1998; Sinervo & Lively, 1996). Orange–blue males pursue the strategy of being an aggressive territory holder with a harem of several females, whereas orange males are larger, but less aggressive, non‐territorial cuckolders of the harem holders (Moore et al., 1998).

Alternative male morphs are especially common within teleost fishes (Mank & Avise, 2006; Taborsky, 2008). For example, males of the midshipman fish (*Porichthys notatus*) take one of two alternative reproductive strategies: type‐I males build nests, court females and care for their offspring, whereas type‐II males instead attempt to fertilise eggs by sneaking into the nests of type‐I males (Bass, 2023, 2024). Type‐II males mature at a younger age and have smaller bodies but with relatively larger testes at sexual maturity (Bass, 2023, 2024). Similarly, in the shell‐dwelling cichlid *Lamprologus callipterus*, males of the large ‘nest male’ morph defend collections of gastropod shells in which the much smaller females spawn and care for young (Sato, 1994; von Kuerthy & Taborsky, 2016). Males of the ‘dwarf’ morph live within shells like females, never growing to the size of the nesting male, and attempt to parasitise fertilizations from the nest male (Taborsky, 2001; Wirtz Ocana et al., 2014). Alternative male morphs may be relatively common in teleost fishes because most teleosts show indeterminate growth allowing for substantial variation in adult body size. Differences in adult body size are often a characteristic of alternative male morphs and show flexibility in ontogeny and sexual differentiation, which may open additional avenues for phenotypic variation (Oliveira, 2005).

Alternative male morphs often show strikingly distinct behavioural profiles and reproductive tactics (Knapp & Neff, 2008; Mank & Avise, 2006; Taborsky, 2001, 2008). The association between male morph and behaviour suggests variation between morphs in underlying neuroendocrine mechanisms (Bass, 1992; Bass, 2024; Brantley et al., 1993; Giraldo‐Deck et al., 2024; Godwin, 2010; Lipshutz et al., 2019; Oliveira, 2005). For example, in midshipman males, both morph and reproductive tactic are associated with differential expression of at least six neurohormones in the preoptic area (POA) of the hypothalamus (galanin, urocortin‐3‐like, corticotropin‐releasing hormone precursor, oxytocin receptor, thyrotropin subunit beta and growth hormone; Feng & Bass, 2017; Tripp et al., 2018, Tripp et al., 2020).

The nonapeptides oxytocin (OXT) and vasotocin (AVT) are known to be involved in regulating a wide range of social behaviours, including aggression and social attraction (Albers, 2015; Dumais & Veenema, 2016; Goodson & Thompson, 2010), although the role of these peptides in regulating social behaviour in fishes is less well established than in mammals (Godwin & Thompson, 2012; Kareklas et al., 2025). In the brains of teleost fishes, OXT (often referred to as isotocin in teleosts) and AVT are produced exclusively within three populations of cells in the POA (Goodson & Bass, 2001; Godwin & Thompson, 2012; Thompson & Walton, 2013; Huffman et al., 2012; Banerjee et al., 2017). These cells can be discriminated based on their size, location and cytoarchitecture. Parvocellular cells are the smallest and are located rostro‐ventrally within the POA. Caudo‐dorsally from the parvocellular cells are the larger magnocellular cells. The gigantocellular cells are the furthest dorsal, largest and, in contrast to the parvo‐ and magnocellular cells, are consistently multipolar (Almeida & Oliveira, 2015; Bradford & Northcutt, 1983; Fernald & Shelton, 1985; Godwin & Thompson, 2012). Each of these cell populations projects to the pituitary, as well as diverse targets throughout the brain, including the hindbrain (Thompson & Walton, 2009), thalamus (Saito et al., 2004) and ventral telencephalon (Maruska, 2009; Saito et al., 2004). The number and/or size of nonapeptide‐producing neurons in the POA has been associated with social behaviour in fishes (Dewan et al., 2008; Dewan et al., 2011; Dewan & Tricas, 2011; Foran & Bass, 1998; Grober et al., 2002; Larson et al., 2006; Lema, 2006; Miranda et al., 2003). AVT typically correlates with aggression and dominance (Almeida & Oliveira, 2015; Greenwood et al., 2008; Lema et al., 2015; Ruberto et al., 2025), whereas OXT is more often related to prosocial behaviour, affiliation and subordinance (Akinrinade et al., 2023; Gemmer et al., 2022; Hellmann et al., 2015; Nunes et al., 2021; O'Connor et al., 2016; Reddon et al., 2012; Teles et al., 2016). These behaviours often differ between alternative male morphs (Taborsky, 2001), and, therefore, the nonapeptides may be a good candidate for the mechanistic underpinning morph‐typical behaviour (Foran & Bass, 1998; Godwin, 2010; Goodson & Bass, 2000; Grober et al., 2002; Semsar et al., 2001).

Males of the West African cichlid *Pelvicachromis pulcher* (commonly known as the rainbow krib) are found in four distinct colour morphs: red, yellow, blue and green, named for the colour of their opercula (Heiligenberg, 1965). The two most common, and best studied, of these are the red and yellow morphs (see Figure S1 for example images), which show differences in reproductive tactics, territoriality and aggression (Martin & Taborsky, 1997; Reddon & Hurd, 2013; Seaver & Hurd, 2017). In a naturalistic environment, about half of red males became harem holders and the rest formed monogamous pairs, whereas about a third of yellow males bred monogamously, and the rest were satellite males associated with the territories of dominant harem holding red males (Martin & Taborsky, 1997). No red males were observed to function as satellite males, nor were yellow males observed to hold a harem even in the absence of a competitors (Martin & Taborsky, 1997). Red males have higher gonadosomatic index (GSI) and are more aggressive than yellow males (Seaver & Hurd, 2017). Previously, it was suggested, based on observations of a small number of spawning events in the laboratory, that the basis for these different morphs may be genetic, and that male morphs could be fixed for life (Heiligenberg, 1965). However, more recently, there is evidence that both sex and male morphs in *P. pulcher* are influenced by the pH of the water in their environment during early life such that lower pH produces a more male‐biased sex ratio and a higher proportion of red males relative to yellow males compared to water at a higher pH (Reddon & Hurd, 2013; Rubin, 1985).

Here we examine the differences in size and number of AVT‐ and OXT‐producing cells in the POA of both red and yellow *P. pulcher* males while controlling for body size, territorial status and social dominance using standardised laboratory housing conditions. We predict that the more aggressive red males will have larger and/or more numerous AVT neurons than the yellow males, whereas the more submissive and gregarious yellow males will have larger and/or more numerous OXT neurons.

## 2 MATERIALS AND METHODS

### 2.1 Subjects and housing

The study was conducted at the University of Alberta (Canada) in 2015–2016. Adult *P. pulcher* were obtained from a local commercial supplier and housed briefly together in stock aquaria. Fish were then moved into 38‐L aquaria divided into six equal compartments with transparent plastic barriers. Three males and three females were placed into compartments such that each focal male inhabited a compartment adjacent to at least one male and one female for a period of at least 10 weeks. We assigned fish to compartments based only on sex and not male morph, which was determined by observation after the social experience phase. Each fish was provided with a piece of terracotta pot for use as a shelter. This set‐up served to standardise the recent social experience between red and yellow males prior to tissue collection. Water temperature was maintained at 25 ± 1°C, and housing rooms were kept on a 12:12‐h light: dark cycle. Fish were fed once a day ad libitum 5 days a week with dried prepared flake food or freeze‐dried brine shrimp. For further details of the housing and husbandry regime, see Seaver and Hurd (2017).

Ten males from each morph (total *n* = 20) were identified based on visual inspection of their opercula with red males displaying conspicuous red colouration, whereas yellow males showed a more muted yellow colour. Prior to tissue collection, fish were weighed and measured for standard length.

### 2.2 Tissue collection

Subjects were irreversibly anaesthetized by immersion in an overdose of tricaine methanesulfonate followed by rapid decapitation. The heads were then placed in 4% paraformaldehyde (PFA) solution for 24 h. Brains were then extracted and post‐fixed in 4% PFA overnight and transferred to 30% sucrose in 0.1 M phosphate‐buffered saline (PBS) pH of 7.4 and kept at 4°C until they sunk. We dissected out and weighed the gonads from each male in the sample.

### 2.3 Immunohistochemistry

Cryoprotected brains were embedded in a 12% w/v gelatine. Excess gelatine was then trimmed, and the remaining block was placed in 4% PFA post‐fix for 2–5 h. Gelatine blocks were then transferred to a 30% sucrose in 0.1 M PBS solution and kept at 4°C overnight. Brains were then sectioned at 25 μm in the coronal plane using a freezing microtome, and the tissue was collected in PBS and 1% sodium azide. Sections were then transferred to well plates for further processing. On day 1, floating sections were rinsed 3 times in PBS (5 m each) and blocked for 1 h in 10% normal donkey serum (Cederlane Labs, Burlington, Ontario, Canada) and 0.1% Triton X‐100 in 0.1 M PBS. Tissue was then incubated for 1 day at 4°C in PBS with 0.1% Triton X‐100, 2.5% normal donkey serum and both rabbit anti‐AVT (catalogue number: T‐4563) and guinea pig anti‐OXT (catalogue number: T‐5021) (1:5000, Peninsula Laboratories, California, USA), which have previously been used in African cichlids (Reddon et al., 2017). After this, floating sections were rinsed five times in PBS (5 min each) and incubated for 2 h at room temperature in PBS, with 0.1% Triton X‐100, 2.5% normal donkey serum and both donkey anti‐rabbit Alexa Fluor 488 (catalogue number: 711‐005‐152) and donkey anti‐guinea pig Alexa Fluor 594 (catalogue number: 706‐005‐148) (1:200, Jackson ImmunoResearch Laboratories Inc., Pennsylvania, USA) secondary antibodies. Floating sections were then thoroughly washed five times in PBS (5 m each), mounted in low light conditions on gelatinized glass slides and left to air‐dry at 4°C in a dark environment. Three fish (one red male and two yellow males) were removed from the final sample due to excess tissue loss during sectioning or staining (final *n* = 17).

### 2.4 Microscopy and cell quantification

The sections were viewed using a compound light microscope (Leica DMRE) equipped with the appropriate fluorescence filters for visualisation at 20× magnification. An average of 39 sections per individual contained nonapeptide neurons (range 15–55). Images were acquired using a Retiga EXi FAST Cooled Mono 12‐bit camera (QImaging) and analysed with Openlab imaging software (Improvision). See Figure S2 for sample images.

Cell counting and measurements were completed blind to male morph. Cells were classified as parvocellular, magnocellular or gigantocellular based on neuroanatomical location, size and morphology (based on Bradford & Northcutt, 1983; Almeida & Oliveira, 2015). Cell counts and measurements were completed using ImageJ 1.50b (National Institutes of Health, USA). Cell size measurements were carried out by tracing the perimeter of the cell soma of a randomly selected (via random number generator) nonpeptide immunoreactive cell body in each section and then averaging for each cell type (parvo, mangno and gigantocellular for OXT and AVT) within each individual.

### 2.5 Statistical analysis

Body mass and standard length were compared between red and yellow males using Welch's *t*‐tests. The size of the parvo, magno and gigantocellular neurons for both OXT and AVT were analysed as dependent variables, with male morph as a categorical independent variable and with body mass included as continuous covariate using a multivariate analysis of covariance (MANCOVA). A second MANCOVA analysis was conducted with the cell count from each cell type and nonapeptide as a dependent variable and male morph as an independent variable, with body mass as a continuous covariate. Models were checked for the assumption of homogeneity of variance using Levene's tests. The measured variables showed no significant heterogeneity of variance between red and yellow males (all *p* > 0.19) apart from the count of parvocellular OXT cells (*p* = 0.008), which was then additionally confirmed using a Welch's *t*‐test. Adherence to the assumption of normally distributed residuals was verified using Shapiro–Wilks tests and by visual inspection of Q–Q plots. No significant departures from normality were detected (all *p* > 0.22). All values are reported as mean ± standard deviation (SD). All analysis was performed using SPSS version 29 for Macintosh.

## 3 RESULTS

### 3.1 Body and gonad size

There was no significant difference between red and yellow males in mass (yellow: 2.65 ± 0.68 g; red: 2.36 ± 0.19 g; Welch's *t*_8.01_ = 1.15, *p* = 0.28) or standard length (yellow: 4.55 ± 0.30 cm; red: 4.39 ± 0.12 cm; Welch's *t*_8.90_ = 1.44, *p* = 0.09). There was no significant difference between red and yellow males in gonad mass (yellow: 0.0013 ± 0.0009 g; red: 0.0014 ± 0.0005 g; Welch's *t*_7.53_ = 0.18, *p* = 0.86).

### 3.2 Cell size

**FIGURE 1:**
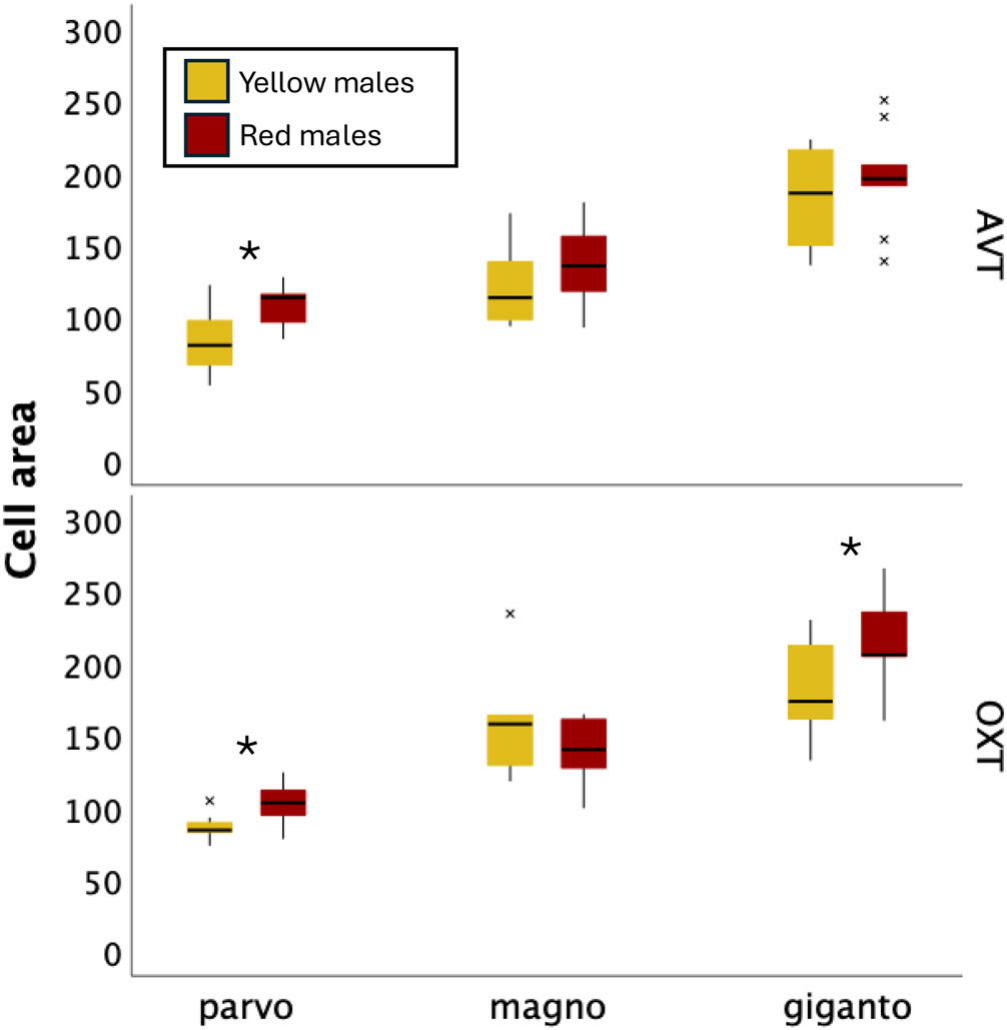
Boxplots representing the cell area (μm^2^) for parvocellular, magnocellular and gigantocellular vasotocin (AVT, top row) and oxytocin cells (OXT, bottom row) from yellow morph males (yellow boxes; *n* = 8) and red morph males (red boxes; *n* = 9) of the cichlid fish *Pelvicachromis pulcher*. Red males have larger parvocellular AVT (*p* = 0.02) and OXT cells (*p* = 0.046), as well as gigantocellular OXT cells (*p* < 0.01). Significant differences (*p* < 0.05) are indicated by an asterisk. Boxes represent medians ± interquartile range (IQR).

Red males had significantly larger parvocellular AVT and OXT cells, as well as larger gigantocellular OXT cells (Table 1; Figure 1). There was no significant difference in the size of gigantocellular AVT cells between the morphs, nor in the size of magnocellular cells for either nonapeptide (Table 1; Figure 1).

**TABLE 1:** Results from a multivariate analysis of covariance (MANCOVA) comparing cell sizes between red and yellow males of the cichlid fish *Pelvicachromis pulcher* for parvocellular, magnocellular and gigantocellular cells for oxytocin (OXT) and vasotocin (AVT) cells in the preoptic area, including body mass as a covariate.

Nonapeptide	Cell type	Yellow male cell area (μm^2^; mean ± SD)	Red male cell area (μm^2^; mean ± SD)	*F*_(1,14)_	*p*
**AVT**	**parvo**	**82.94 ± 22.64**	**108.74 ± 13.70**	**7.64**	**0.015**
AVT	magno	137.47 ± 29.31	120.54 ± 29.11	0.55	0.471
AVT	giganto	196.19 ± 35.36	182.66 ± 34.96	0.66	0.431
**OXT**	**parvo**	**86.45 ± 9.11**	**101.68 ± 14.76**	**4.80**	**0.046**
OXT	magno	156.93 ± 36.14	139.48 ± 22.24	2.49	0.137
**OXT**	**giganto**	**182.21 ± 33.52**	**215.97 ± 30.20**	**9.98**	**0.007**

*Note*: Significant differences (*p* < 0.05) between red and yellow males are indicated with bold text.

Abbreviation: SD, standard deviation.

### 3.3 Cell count

There was no significant difference between red and yellow males in the number of OXT or AVT cells in the parvocellular, magnocellular or gigantocellular populations (Table 2). Due to heterogeneity of variance, the lack of significant difference between red and yellow males in parvocellular OXT cell number was confirmed by Welch's *t*‐test (*t*_13.02_, *p* = 0.64).

**TABLE 2:** Results from a multivariate analysis of covariance (MANCOVA) comparing cell numbers between red and yellow males of the cichlid fish *Pelvicachromis pulcher* for parvocellular, magnocellular and gigantocellular cells for vasotocin (AVT) and oxytocin (OXT) cells in the preoptic area, including body mass as a covariate.

Nonapeptide	Cell type	Yellow male cell number (mean ± SD)	Red male cell number (mean ± SD)	*F*_(1,14)_	*p*
AVT	parvo	155.50 ± 81.96	149.33 ± 62.06	0.04	0.854
AVT	magno	110.63 ± 47.11	124.89 ± 40.60	0.67	0.426
AVT	giganto	45.50 ± 11.80	45.00 ± 22.20	0.39	0.541
OXT	parvo	289.38 ± 67.54	311.56 ± 117.21	0.46	0.506
OXT	magno	182.63 ± 80.74	180.63 ± 49.83	<0.01	0.968
OXT	giganto	76.75 ± 32.74	70.44 ± 22.99	0.10	0.761

Abbreviation: SD, standard deviation.

## 4 DISCUSSION

We quantified the size and number of AVT‐ and OXT‐producing neurons in the POA of the hypothalamus as a function of male morph in the cichlid fish *P. pulcher*. We predicted larger and/or more numerous AVT cells in the red males and larger and/or more numerous OXT cells in the yellow males. Our predictions were partly supported in the larger parvocellular AVT cells in red males, but surprisingly we also found larger parvo‐ and gigantocellular OXT cells in red males. All males in this study were adults of approximately the same size and were given an exclusive home territory with visual access to both a female and male conspecifics, thereby controlling for recent social experience, which may otherwise differ substantially between red and yellow males (Martin & Taborsky, 1997). As a result, the differences observed in nonapeptide cell size likely reflect stable differences between the red and yellow male morphs, potentially organised early in life.

Prior work in other species of fishes has found that AVT plays a significant role in aggression and dominance. Santangelo and Bass (2006) reported that in the beaugregory damselfish (*Stegastes leucostictus*) the exogenous administration of AVT increased rates of aggression, whereas an AVT receptor antagonist reduced aggression. Ruberto et al. (2025) found that dominant daffodil cichlids (*Neolamprologus pulcher*) have more numerous parvocellular AVT cells compared to subordinates, and fish with higher numbers of AVT cells were also more aggressive. In the same species, dominant fish also had larger AVT cells in all three cell groups, and dominant females with larger AVT cells were more aggressive (Ruberto, 2024). Foran and Bass (1998) reported AVT cell size differences between territorial and non‐territorial plainfin midshipman (*Porichthys notatus*) males, with the non‐territorial sneaker males having smaller AVT cells than the territorial parental males. There was no difference between the midshipman male morphs in the number of AVT cells in the POA, consistent with our results.

Previous studies have found that OXT may be positively related to the expression of prosocial behaviour (Braida et al., 2012; Gemmer et al., 2022; O'Connor et al., 2016; Thompson & Walton, 2004) and social subordination (Hellmann et al., 2015; O'Connor et al., 2016; Reddon et al., 2012; Teles et al., 2016). We predicted that the yellow males would show greater OXT expression fitting with this behavioural phenotype, as they must be motivated to associate closely with conspecifics and able to behave deferentially to succeed as a satellite on a red male's territory (Martin & Taborsky, 1997; Reddon, Ruberto, & Reader, 2022). However, although we did find a difference in the size of parvo and gigantocellular OXT neurons, surprisingly it was the red males that had larger cells on average. Some prior studies have found positive relationships between aggression and OXT. For example, Kleszczyńska et al. (2012) found that free OXT levels in the brain were positively related to male aggression in the three‐spined stickleback. OXT may also be associated with social withdrawal or reduced social motivation in some species or contexts. For example, Reddon et al. (2014) found that exogenous OXT reduced shoaling tendency, whereas an OXT antagonist increased it in *N. pulcher*, and endogenous OXT levels correlated negatively with affiliative behaviour in the same species (Reddon et al., 2015). Reddon et al. (2017) found that cooperatively breeding cichlid fishes had fewer OXT neurons than their less social relatives. Guppies (*Poecilia reticulata*) from high‐predation populations, which exhibit greater shoaling behaviour, do not show greater brain gene expression of OXT nor its central receptors compared to less‐social, low‐predation guppies (Reddon, Aubin‐Horth, & Reader, 2022). In general, species and context differences in the role of OXT on social behaviour are common among fishes (Kareklas et al., 2025), and the perception of OXT as purely prosocial or bonding‐related peptide is a misleading oversimplification (van Anders et al., 2013; Walle et al., 2025). A further complication is that OXT and AVT can bind to each other's receptors in fishes, albeit with a reduced affinity, and therefore some of the seemingly inconsistent results reported in fishes may stem from OXT acting upon central AVT receptors or vice versa (Kareklas et al., 2025).

Our study controls for recent social experience across these two alternative male morphs, something likely to be confounded using a more naturalistic set‐up (e.g., Martin & Taborsky, 1997). Individual differences in neuronal phenotype can stem from plastic changes reflecting recent social or environmental experience or from relatively stable differences organised during development (Thompson & Walton, 2013). The differences between red and yellow males that we detected appear to be robust to the effects of recent experience. It is possible that the differences in nonapeptide cell size between red and yellow males are organised by the sexual differentiation process during development. The pH of the water early in life affects both the ratio of males to females and of red to yellow males (Reddon & Hurd, 2013). Yellow males are relatively more common in female‐biasing developmental conditions, and red males are more common in male‐biasing conditions (Reddon & Hurd, 2013), suggesting that the red males may be a more masculinised male phenotype. If red and yellow males do represent a continuum of masculinization, we predict that females would exhibit neuronal phenotypes more like yellow males than red males. Future work should examine the size of the nonapeptide‐producing cells in the POA of female *P. pulcher*.

This work adds to the growing literature linking nonapeptides to social behaviour and behaviour‐relevant phenotypes in teleost fishes. Our findings are consistent with the established role of AVT in promoting aggressive behaviour and dominance, and with the more nuanced role of OXT in regulating social behaviour in fishes, which may depend on species and social context.

## AUTHOR CONTRIBUTIONS

Peter L. Hurd conceived the study and secured funding. Douglas R. Wylie oversaw the laboratory work. Adam R. Reddon analysed the data. Adam R. Reddon and Peter L. Hurd wrote the manuscript.

## FUNDING INFORMATION

Funding for this project was provided by the Natural Sciences and Engineering Research Council of Canada (NSERC) [NSERC Discovery Grant: RGPIN 249685] to Peter L. Hurd.

## CONFLICT OF INTEREST STATEMENT

The authors declare no competing interests.

## Supporting information


**Data S1.** Supporting information.

**Figure S1.** Example images of red (above) and yellow (below) *Pelvicachromis pulcher* males.
**Figure S2.** Exemplars of vasotocin (AVT) and oxytocin (OXT) parvocellular, magnocellular and gigantocellular cells visualised using immunohistochemistry and fluorescent microscopy. Scale bar = 100 μm. Top row = parvocellular, middle row = magnocellular, bottom row = gigantocellular. Left column = AVT cells, middle column = AVT and OXT cells, right column = OXT cells. AVT cells are indicated in green, and OXT cells are indicated in red.

## Data Availability

The data that supports the findings of this study are available in the supplementary material of this article.
