# Supplementary material for: Nonapeptide cell size differs between male morphs of the West African cichlid, Pelvicachromis pulcher
Source: J Fish Biol. 2025 Nov 27;108(5):1483–90. doi: 10.1111/jfb.70306 (PMC13273080; doi:10.1111/jfb.70306)
Supplement: Supplementary file 2 — Figure S1. Example images of red (above) and yellow (below) Pelvicachromis pulcher males. Figure S2. Exemplars of vasotocin (AVT) and oxytocin (OXT) parvocellular, magnocellular and gigantocellular cells visualised using immunohistochemistry and fluorescent microscopy. Scale bar = 100 μm. Top row = parvocellular, middle row = magnocellular, bottom row = gigantocellular. Left column = AVT cells, middle column = AVT and OXT cells, right column = OXT cells. AVT cells are indicated in green, and OXT cells are indicated in red. [file JFB-108-1483-s002.docx]

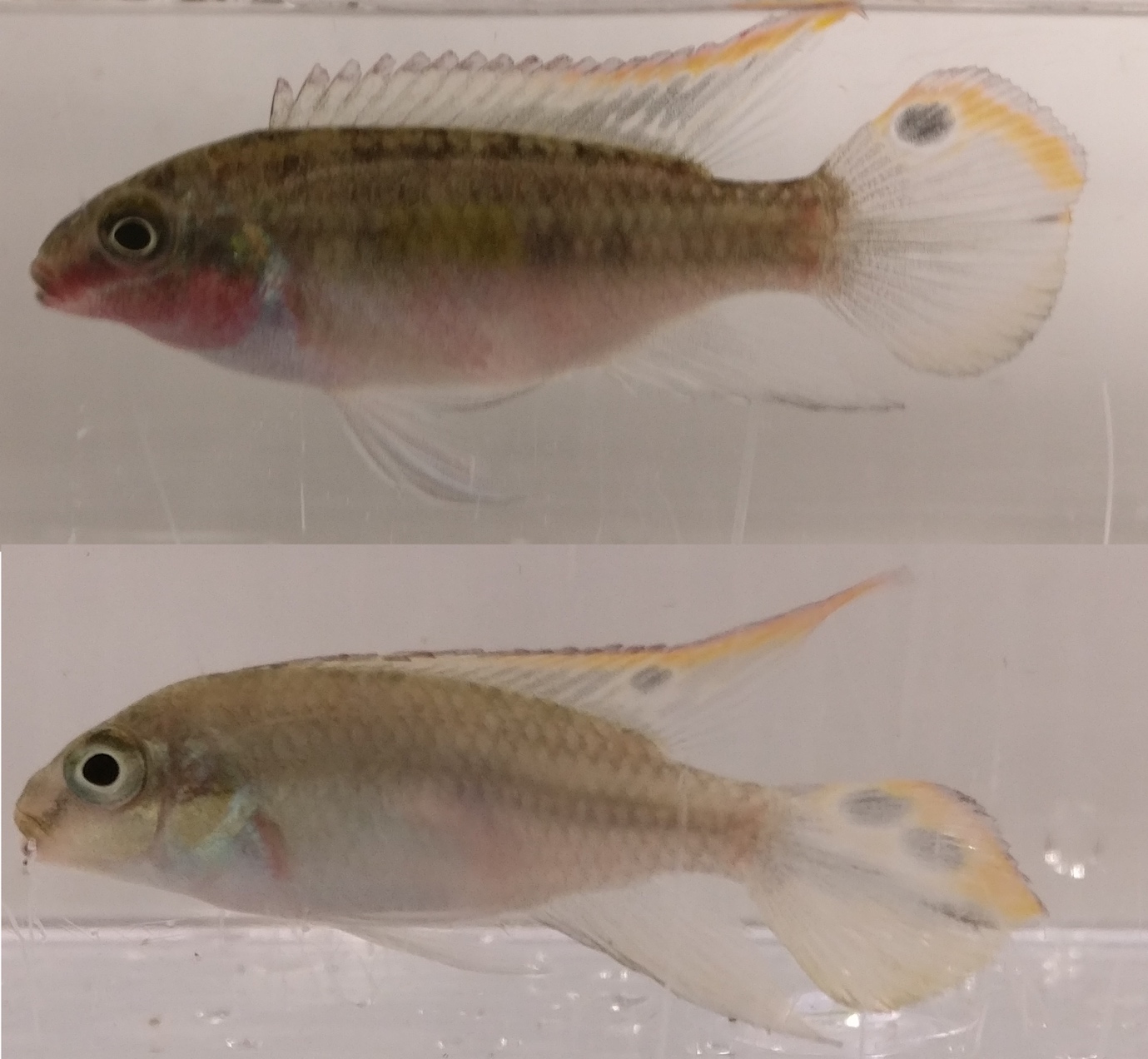


**Figure S1.** Example images of red (above) and yellow (below) *Pelvicachromis pulcher* males.


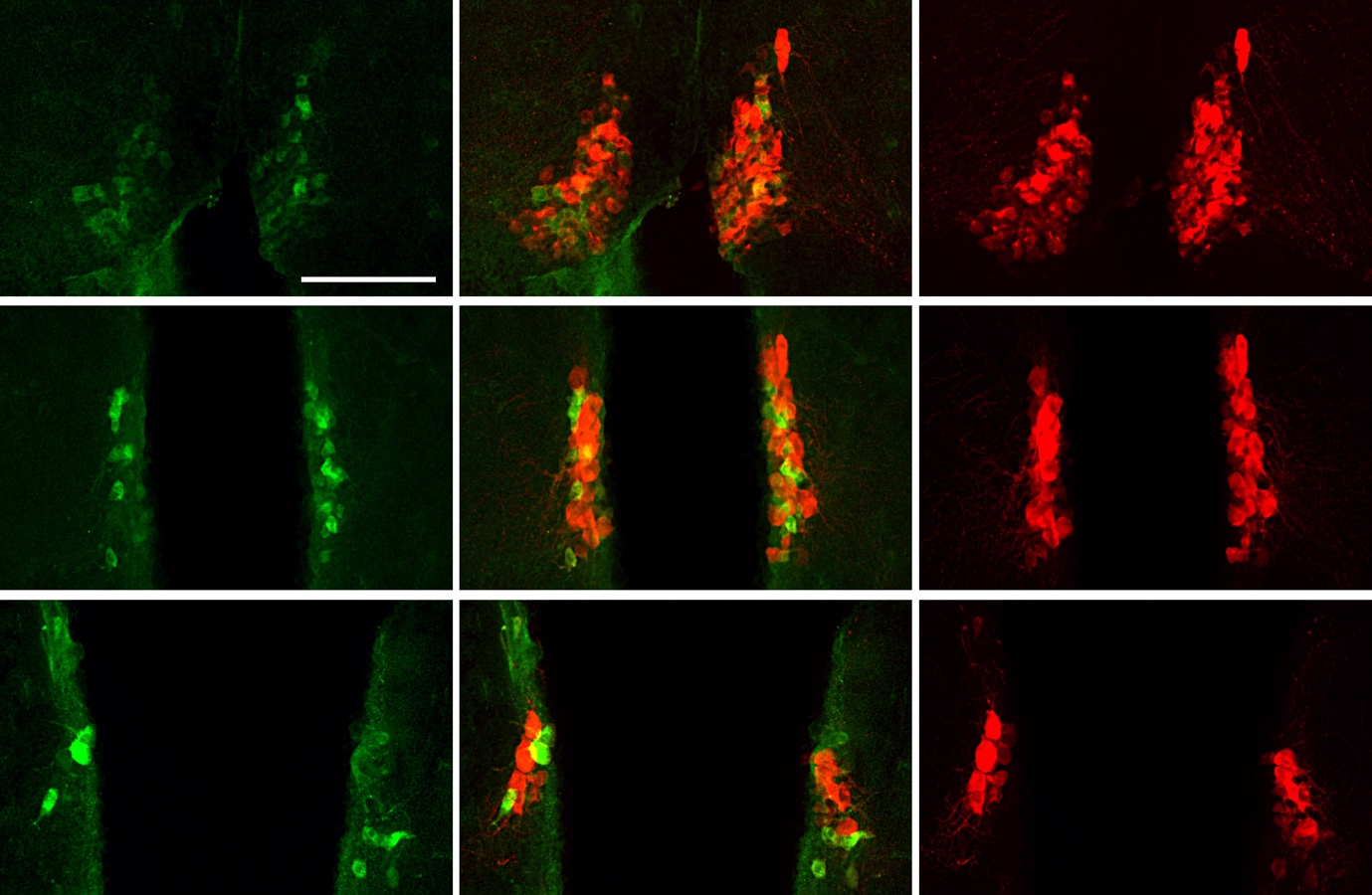


**Figure S2.** Exemplars of AVT and OXT parvocellular, magnocellular, and gigantocellular cells visualised by immunohistochemistry and fluorescent microscopy. Scale bar = 100µm. Top row = parvocellular, middle row = magnocellular, bottom row = gigantocellular. Left column = AVT cells, middle column = AVT and OXT cells, right column = OXT cells. AVT cells are in green, OXT cells are in red.
